# Exercise Training Attenuates Hypertension and Cardiac Hypertrophy by Modulating Neurotransmitters and Cytokines in Hypothalamic Paraventricular Nucleus

**DOI:** 10.1371/journal.pone.0085481

**Published:** 2014-01-17

**Authors:** Lin-Lin Jia, Yu-Ming Kang, Fu-Xin Wang, Hong-Bao Li, Yan Zhang, Xiao-Jing Yu, Jie Qi, Yu-Ping Suo, Zhen-Jun Tian, Zhiming Zhu, Guo-Qing Zhu, Da-Nian Qin

**Affiliations:** 1 Department of Physiology and Pathophysiology, Xi’an Jiaotong University Cardiovascular Research Center, Xi’an Jiaotong University School of Medicine, Xi’an, China; 2 Department of Physiology, Shantou University Medical College, Shantou, China; 3 Department of Neurology, The First Affiliated Hospital of Jiamusi University, Jiamusi, China; 4 Department of Hypertension and Endocrinology, Center for Hypertension and Metabolic Diseases, Daping Hospital, The Third Military Medical University, Chongqing Institute of Hypertension, Chongqing, China; 5 Institute of Sports and Exercise Biology, School of Physical Education, Shaanxi Normal University, Xi’an, China; 6 Department of Obstetrics and Gynecology, Shanxi Provincial People’s Hospital, Taiyuan, China; 7 Key Laboratory of Cardiovascular Disease and Molecular Intervention, Department of Physiology, Nanjing Medical University, Nanjing, China; Universidad Europea de Madrid, Spain

## Abstract

**Aims:**

Regular exercise as an effective non-pharmacological antihypertensive therapy is beneficial for prevention and control of hypertension, but the central mechanisms are unclear. In this study, we hypothesized that chronic exercise training (ExT) delays the progression of hypertension and attenuates cardiac hypertrophy by up-regulating anti-inflammatory cytokines, reducing pro-inflammatory cytokines (PICs) and restoring the neurotransmitters balance in the hypothalamic paraventricular nucleus (PVN) in young spontaneously hypertensive rats (SHR). In addition, we also investigated the involvement of nuclear factor-κB (NF-κB) p65 and NAD(P)H oxidase in exercise-induced effects.

**Methods and results:**

Moderate-intensity ExT was administrated to young normotensive Wistar-Kyoto (WKY) and SHR rats for 16 weeks. SHR rats had a significant increase in mean arterial pressure and cardiac hypertrophy. SHR rats also had higher levels of glutamate, norepinephrine (NE), phosphorylated IKKβ, NF-κB p65 activity, NAD(P)H oxidase subunit gp91^phox^, PICs and the monocyte chemokine protein-1 (MCP-1), and lower levels of gamma-aminobutyric acid (GABA) and interleukin-10 (IL-10) in the PVN. These SHR rats also exhibited higher renal sympathetic nerve activity (RSNA), and higher plasma levels of PICs, and lower plasma IL-10. However, ExT ameliorates all these changes in SHR rats.

**Conclusion:**

These findings suggest that there are the imbalances between excitatory and inhibitory neurotransmitters and between pro- and anti-inflammatory cytokines in the PVN of SHR rats, which at least partly contributing to sympathoexcitation, hypertension and cardiac hypertrophy; chronic exercise training attenuates hypertension and cardiac hypertrophy by restoring the balances between excitatory and inhibitory neurotransmitters and between pro- and anti-inflammatory cytokines in the PVN; NF-κB and oxidative stress in the PVN may be involved in these exercise-induced effects.

## Introduction

It is well-known that hypertension is a major risk factor for cardiovascular diseases. Hypertension is a chronic inflammatory state, and cardiac dysfunction and hypertrophy are prevalent in hypertensive patients and animals. A great deal of evidence shows that pro-inflammatory cytokines (PICs) are involved in the hypertensive effect and of prognostic significance [1], [2]. In addition, imbalance neurotransmitters activities in the PVN and excess amount of free radicals are both observed in hypertension and contribute to the progression of hypertension. More importantly, there is an interaction between neurotransmitters and PICs and positive feedback mechanism among PICs, enhanced oxidative stress in the PVN which play a key role in sympathetic regulation of blood pressure [3], [4]. So we hypothesized that the interaction among these factors may be closely associated with the central mechanisms of hypertension.

Exercise training (ExT) has been considered as a non-pharmacological therapeutic strategy substitute for hypertensive patients and recommended by a number of organizations and agencies. A growing body of evidence indicates that ExT helps to improve the quality of life in patients with hypertension [5], [6]. Recent work provides evidences about the anti-hypertensive effect of ExT such as attenuating sympathoexcitation and peripheral levels of PICs and NE [4], [7], [8], but the exact central mechanisms are still unknown.

The present study was undertaken to determine whether ExT delays progression of hypertension and attenuates cardiac hypertrophy in spontaneously hypertensive rats by restoring the balances between the excitatory and inhibitory neurotransmitters and between pro- and anti-inflammatory cytokines in the PVN. Our findings provide further evidence and insight for the role of ExT on hypertension and cardiac hypertrophy.

## Materials and Methods

### Ethics Statement

All animal experimental procedures in this study were approved by the Animal Care and Use Committees of Xi’an Jiaotong University and were conducted in accordance with the US National Institutes of Health Guide for the Care and Use of Laboratory Animals.

### Animals and General Experimental Protocol

The 7 week old male normotensive Wistar-Kyoto (WKY) and spontaneously hypertensive rats (SHR) were used in this study. The WKY and SHR rats were separated into the sedentary group (SHYsed and WKYsed) and the exercise group (SHRex and WKYex) at random [4]. Rats in exercise groups were subjected to moderate-intensity exercise (about 60% of maximal aerobic velocity, 5 days per week, 60 min per day at 18 m/min, 0^o^ inclination) on a motor-driven treadmill continuously for a period of 16 weeks [4].

### Measurement of Mean Arterial Pressure

Blood pressure was determined by a tail-cuff occlusion method [9]. Rats were allowed to habituate to this procedure for 3 days prior to each experiment. Blood pressure values were averaged from six consecutive cycles per day obtained from each rat.

### Collection of Blood and Tissue Samples

Rats were decapitated under anesthesia, and then trunk blood and tissue samples were collected. The PVN tissue was isolated following Palkovits’s microdissection procedure as previously described [10], [11]. Plasma and tissue samples were stored at −80°C until assayed.

### Measurement of PVN Tissue Levels of Glutamate, GABA and NE

PVN tissue levels of NE, glutamate and GABA were measured using high-performance liquid chromatography with electrochemical detection (HPLC-EC) as previously described [10]–[13].

### Immunohistochemical Studies

Transverse sections from brains were obtained from the region approximately 1.80 mm from the bregma. Immunohistochemical labeling was performed in floating sections as described previously [11] to identify phosphorylated IKKβ positive neurons and NAD(P)H oxidase subunit gp91^phox^ expression in the PVN. For each animal, the positive neurons within the bilateral borders of the PVN were manually counted in three consecutive sections and an average value was reported.

### Quantification of NF-κB p65 Activity in the PVN

The NF-κB/p65 Active ELISA (Active Motif, USA) kit was used to measure the binding activity of free NF-κB p65 in nuclear extracts using a sandwich ELISA method according to the manufacturer’s instructions.

### Biochemical Assays

The levels of IL-1β, IL-6 and IL-10 in plasma and the levels of TNF-α, IL-1β, IL-6 in PVN tissues were quantified using commercially available rat ELISA kits (Invitrogen) according to manufacturer’s instructions.

### Real-time PCR

Real time PCR amplification reactions were performed to detect the mRNA expression of atrial natriuretic peptide in the left ventricular tissue of the heart, and the mRNA expressions of IL-10 and the MCP-1 in the PVN, as previously described [10], [14]. Data were normalized to GAPDH expression.

### Electrophysiological Recording

Renal sympathetic nerve activity (RSNA) was recorded. The general methods for recording and analyzing RSNA have been described previously [15], [16].

### Statistical Analysis

All data were analyzed by ANOVA followed by a post-hoc Tukey test. Blood pressure data were analyzed by repeated measures ANOVA. Data were expressed as mean ± SEM. A probability value of *P*<0.05 was considered to be statistically significant.

## Results

### Effect of Exercise Training on Mean Arterial Pressure

**Figure 1:**
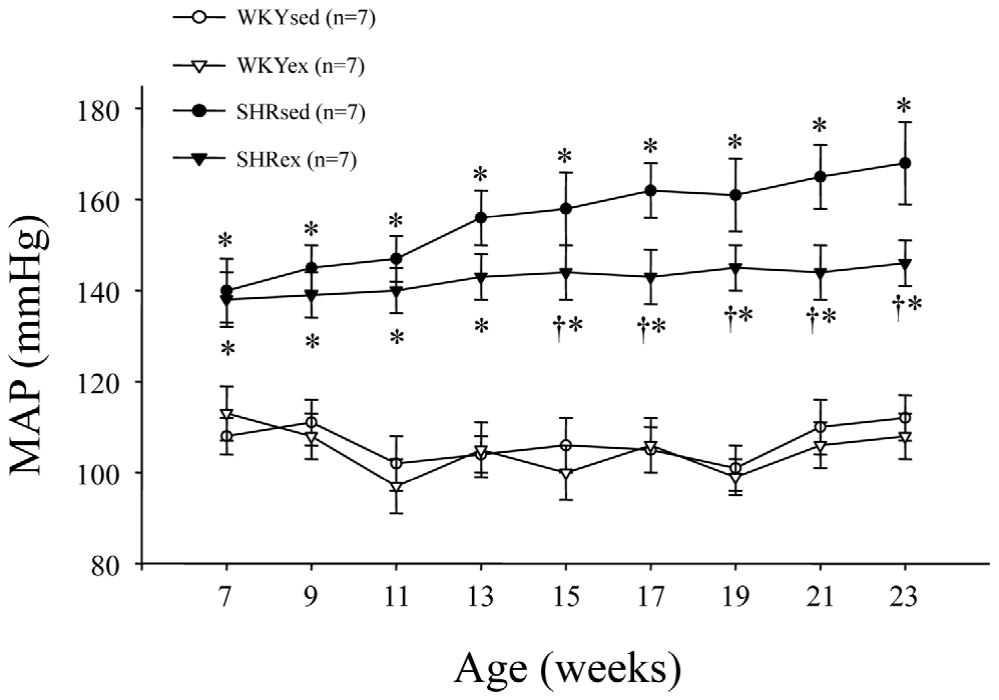
Time course of mean arterial pressure (MAP) in WKY and SHR rats. SHR rats had significant higher MAP when compared with WKY rats. Exercise training reduced MAP in SHR rats from 13 weeks of exercise. *P<0.05 versus control (WKYsed or WKYex); † *P*<0.05 SHRex versus SHRsed.

SHR rats had significant higher mean arterial pressure when compared with WKY rats. Exercise training reduced mean arterial pressure in SHR rats from 13 weeks of exercise, indicating the anti-hypertensive effect of exercise training (Figure 1).

### Effect of Exercise Training on Cardiac Hypertrophy

**Figure 2:**
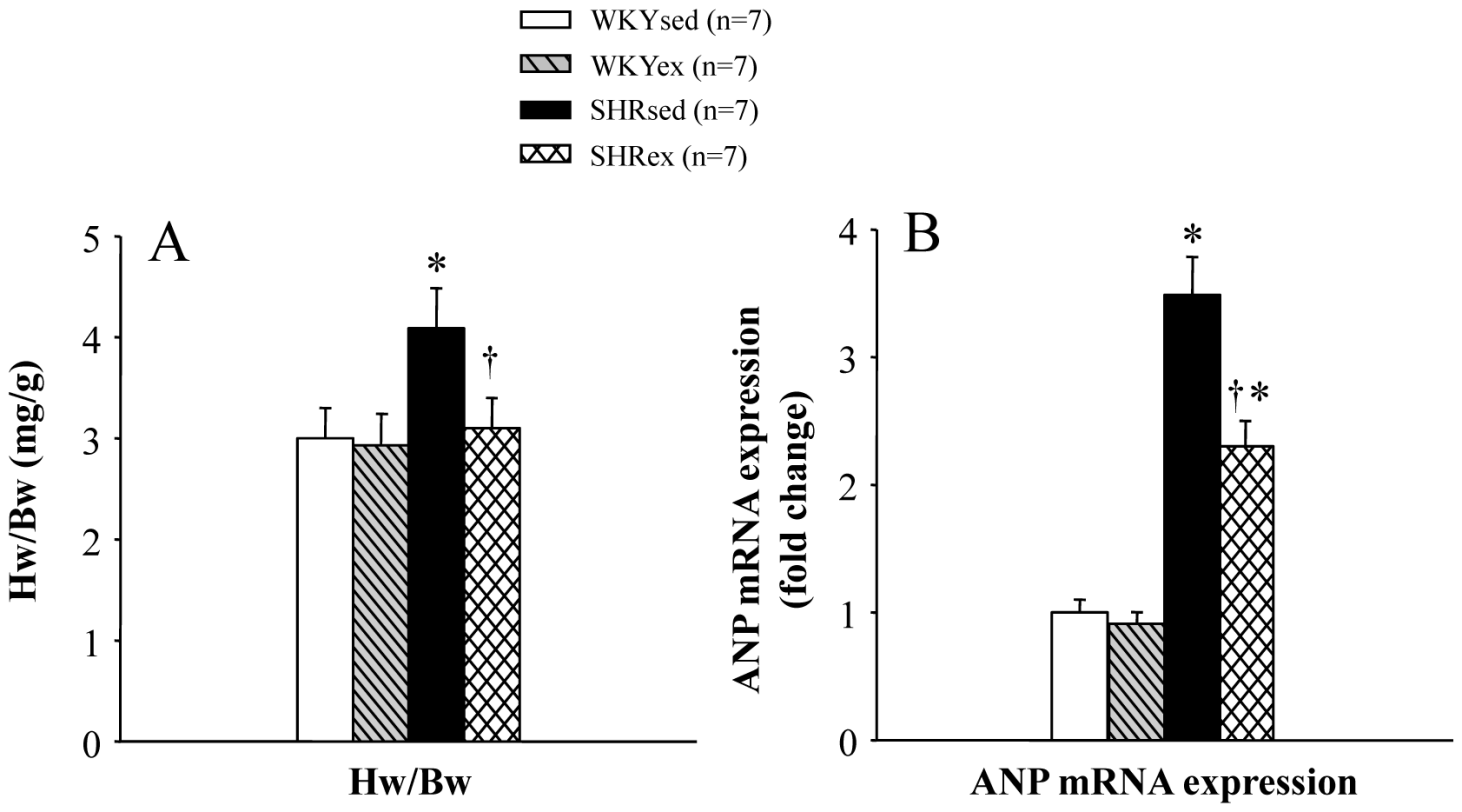
Effect of exercise training on cardiac hypertrophy in WKY and SHR rats. SHR rats had increased cardiac hypertrophy, as assessed by the ratio of heart weight to body weight (Hw/Bw) (A) and mRNA expression of ANP in the left ventricular tissue of the heart (B), which was reduced following exercise training. *P<0.05 versus control (WKYsed or WKYex); † *P*<0.05 SHRex versus SHRsed.

In order to evaluate cardiac hypertrophy, the hearts were harvested and weighed at the end of experiments. The ratio of heart weight/body weight (Hw/Bw) was measured as an indicator of cardiac hypertrophy. SHR rats had increased cardiac hypertrophy, as assessed by the ratio of Hw/Bw (Figure 2A), which was reduced following exercise training. The mRNA expression of a marker of cardiac hypertrophy, atrial natriuretic peptide (ANP), was measured in the left ventricular tissue using real-time PCR. SHR rats had increased mRNA expression of ANP in the left ventricular tissue of the heart, which was decreased by ExT (Figure 2B).

### Effect of Exercise Training on the Neurotransmitters in the PVN

**Figure 3:**
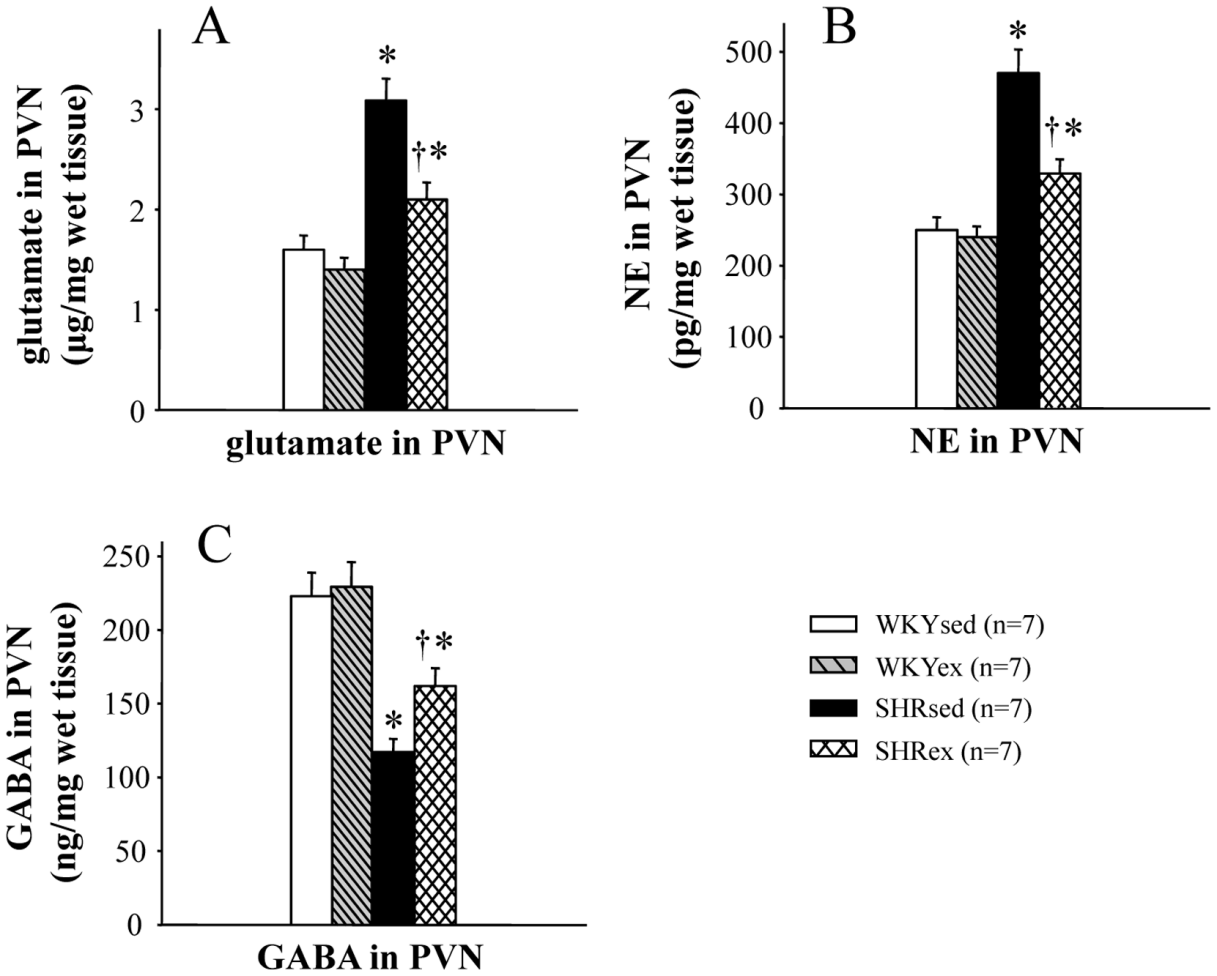
Effect of exercise training on the PVN levels of norepinephrine (NE), glutamate and GABA in WKY and SHR rats. SHR rats had higher levels of glutamate (A) and NE (B), and lower level of GABA (C) in the PVN. Exercise training attenuated the decrease in PVN GABA and the increases in PVN glutamate and NE in SHR rats. *P<0.05 versus control (WKYsed or WKYex); † *P*<0.05 SHRex versus SHRsed.

SHR rats had higher levels of NE and glutamate, and lower level of GABA in the PVN than WKY rats (Figure 3). Exercise training attenuated the decrease in PVN GABA and the increases in PVN glutamate and NE in SHR rats (Figure 3).

### Effect of Exercise Training on NF-κB Activation in the PVN

**Figure 4:**
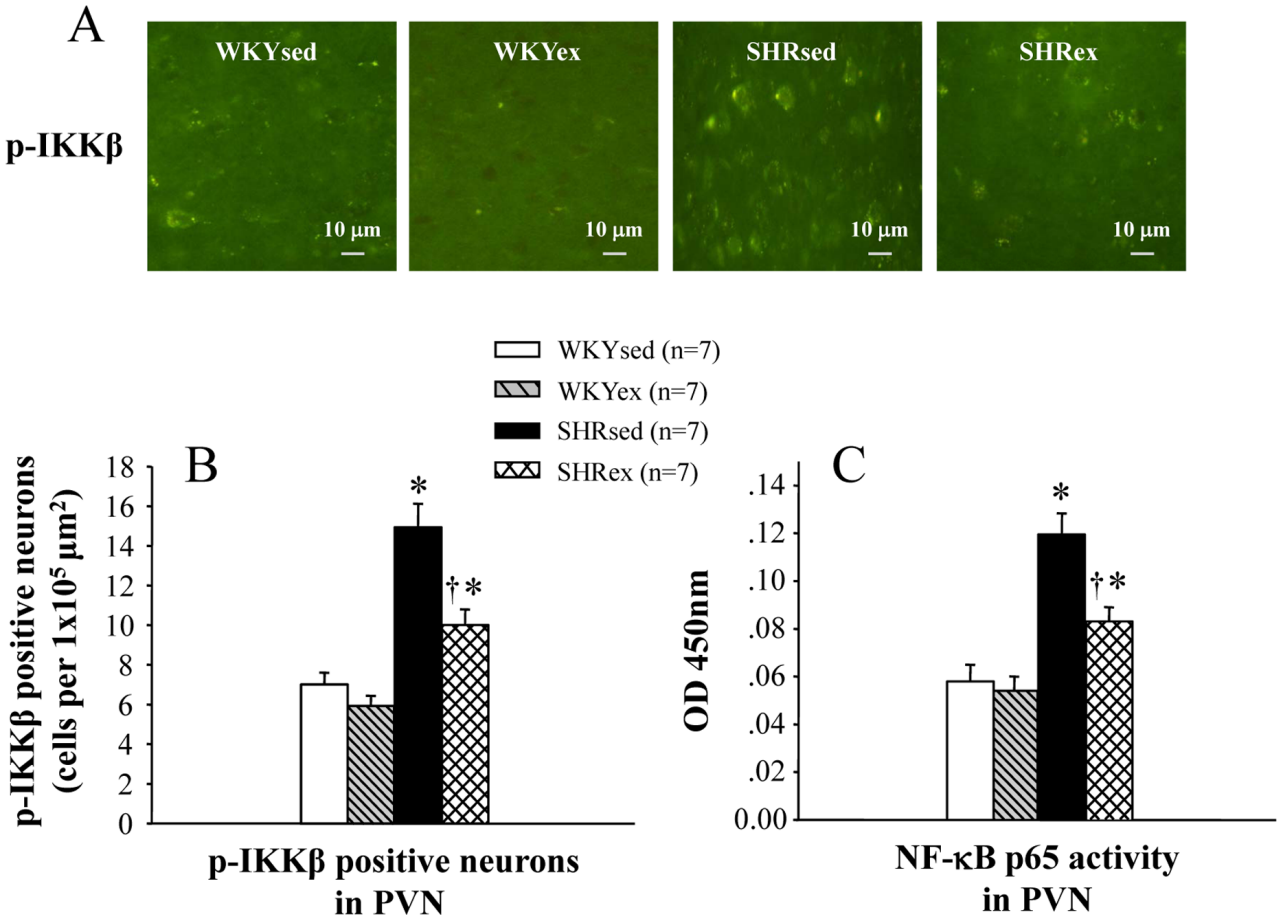
Effects of exercise training on NF-κB p65 activity and the number of p-IKKβ positive neurons in the PVN of WKY and SHR rats. SHR rats showed increases in NF-κB p65 activity and p-IKKβ in the PVN. Exercise training attenuated the increases in NF-κB p65 activity and p-IKKβ in the PVN of SHR rats. *P<0.05 versus control (WKYsed or WKYex); † *P*<0.05 SHRex versus SHRsed.

SHR rats showed increases in NF-κB p65 activity and phosphorylated IKKβ in the PVN. Exercise training attenuated the increases in NF-κB p65 activity and phosphorylated IKKβ in the PVN of SHR rats (Figure 4).

### Effect of Exercise Training on the Number of gp91^phox^ Positive Neurons in the PVN

**Figure 5:**
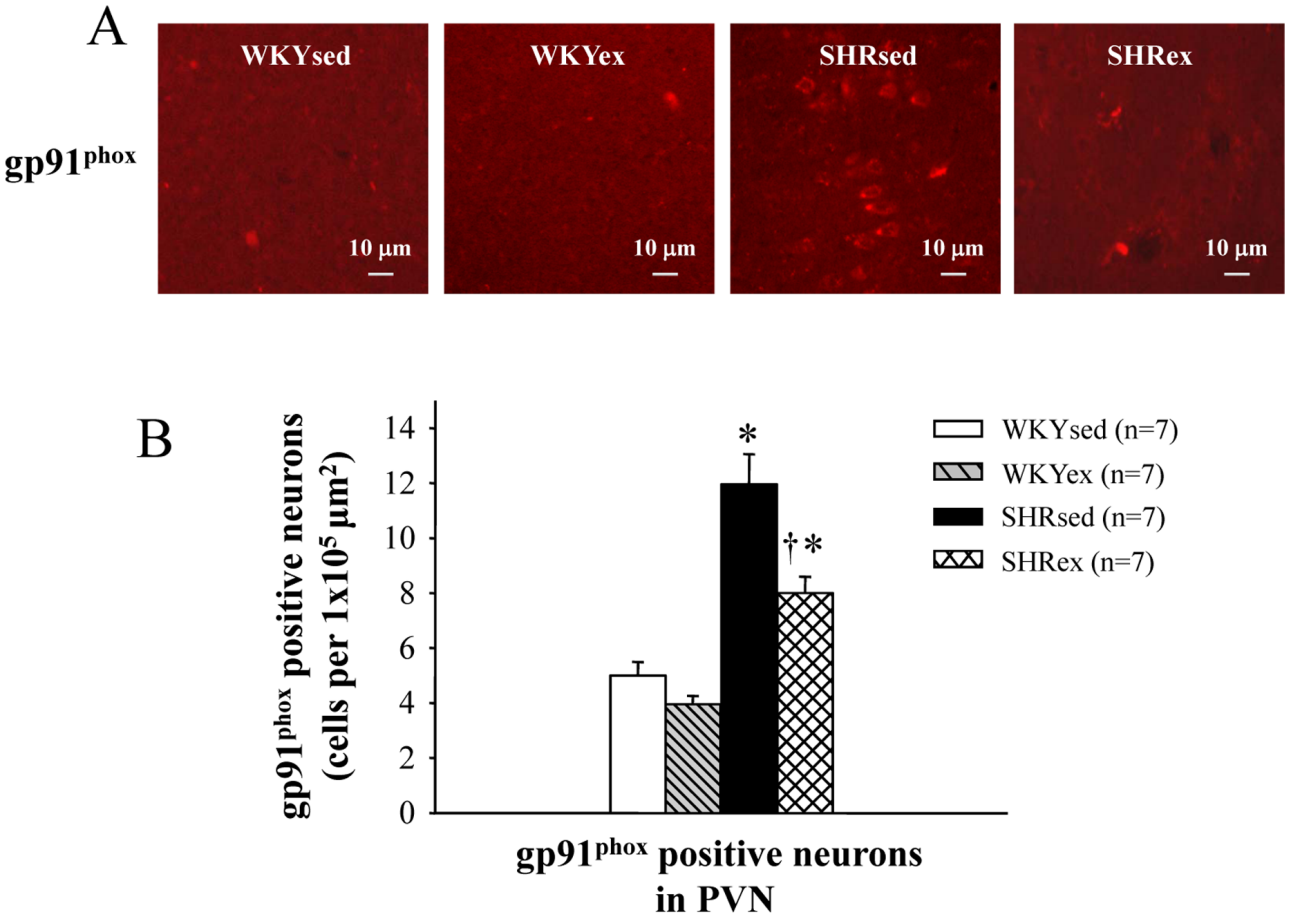
Effect of exercise training on the number of gp91^phox^ positive neurons in the PVN of WKY and SHR rats. SHR rats had more NAD(P)H oxidase subunit gp91^phox^ expression in the PVN than WKY rats. Exercise training decreased gp91^phox^ expression in the PVN in SHR rats. *P<0.05 versus control (WKYsed or WKYex); † *P*<0.05 SHRex versus SHRsed.

SHR rats had more NAD(P)H oxidase subunit gp91^phox^ expression in the PVN than WKY rats. Exercise training decreased gp91^phox^ expression in the PVN of SHR rats (Figure 5).

### Effect of Exercise Training on PICs in the PVN

**Figure 6:**
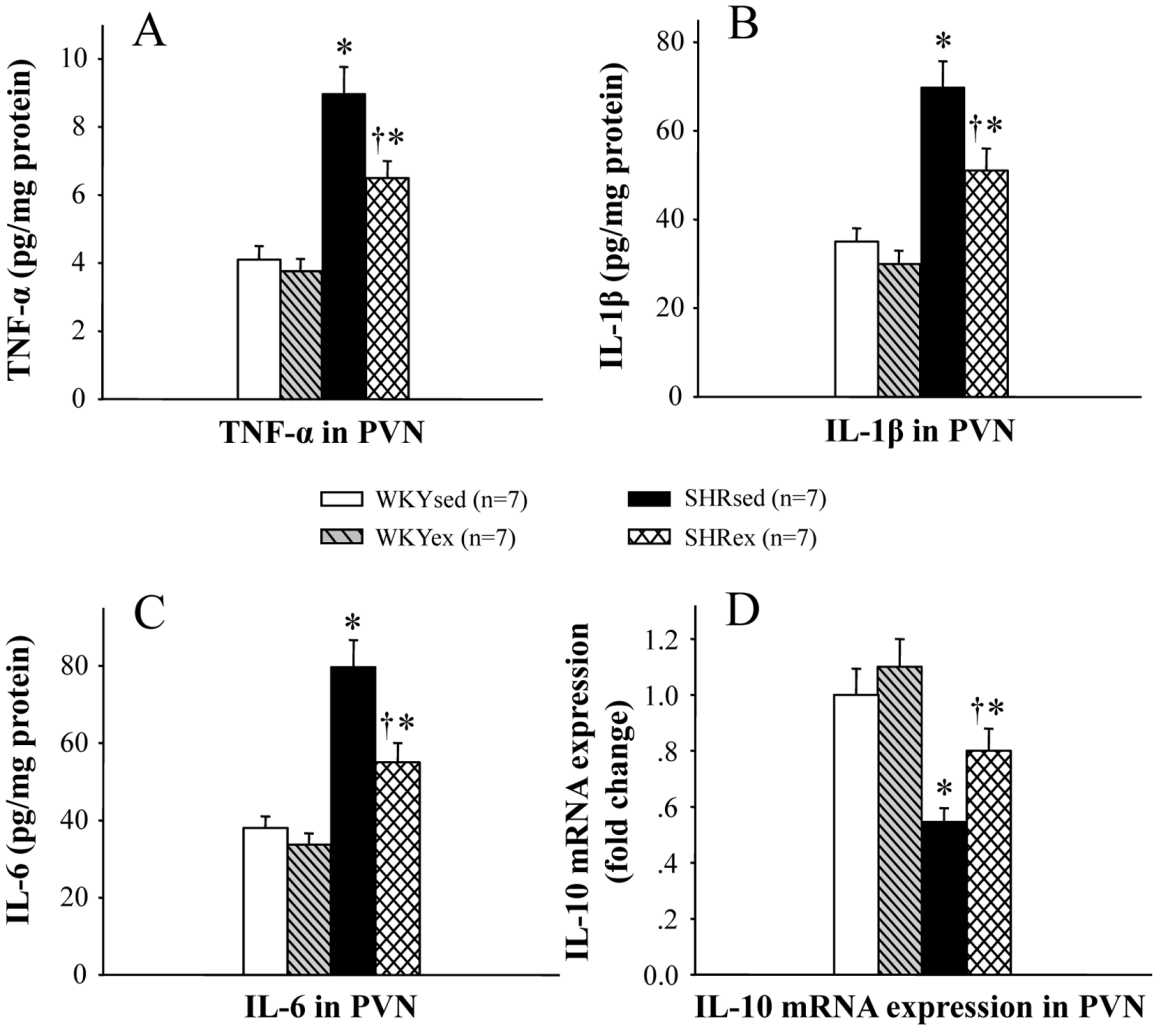
Effect of exercise training on the PVN levels of TNF-α, IL-1β, IL-6 and IL-10 in WKY and SHR rats. PVN levels of TNF-α, IL-1β and IL-6 in SHR rats were higher than in WKY rats, and PVN level of IL-10 in SHR rats was lower than in WKY rats. Exercise training reduced the PVN levels of TNF-α, IL-1β and IL-6 and increased the PVN level of IL-10 in SHR rats. *P<0.05 versus control (WKYsed or WKYex); † *P*<0.05 SHRex versus SHRsed.

**Figure 7:**
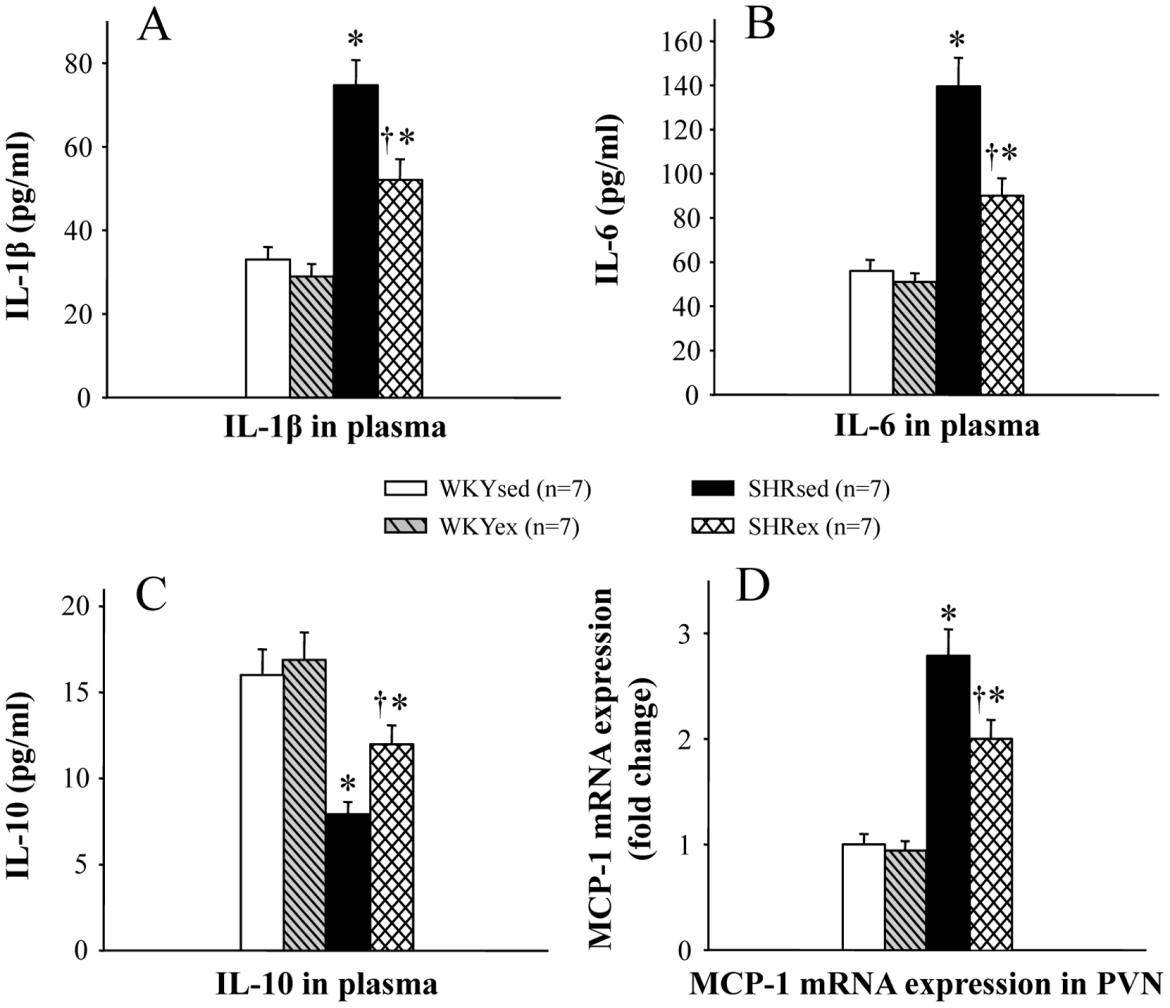
Effect of exercise training on the plasma levels of IL-1β, IL-6 and IL-10 and the PVN level of the chemokine MCP-1 in WKY and SHR rats. Plasma levels of IL-1β and IL-6 and PVN level of the chemokine MCP-1 in SHR rats were higher than in WKY rats, and plasma level of IL-10 in SHR rats was lower than in WKY rats. Exercise training reduced the plasma levels of IL-1β and IL-6 and PVN level of the chemokine MCP-1, and exercise training increased plasma level of IL-10 in SHR rats. *P<0.05 versus control (WKYsed or WKYex); † *P*<0.05 SHRex versus SHRsed.

In order to determine the effect of exercise training on the production of PICs, chemokines and anti-inflammatory cytokines, the levels of TNF-α, IL-1β, IL-6, IL-10 and the MCP-1 were measured in the PVN (Figure 6 and Figure 7D). PVN levels of TNF-α, IL-1β, IL-6 and the MCP-1 in SHR rats were higher than in WKY rats. Exercise training reduced the PVN levels of TNF-α, IL-1β, IL-6 and the MCP-1 in SHR rats. Exercise training restored the balance between pro- and anti-inflammatory cytokines in the PVN of SHR rats.

### Effect of Exercise Training on Plasma Pro- and Anti-inflammatory Cytokines

Plasma levels of IL-1β and IL-6 in SHR rats were higher than in WKY rats, and plasma level of IL-10 in SHR rats was lower than in WKY rats (Figure 7). ExT reduced the plasma levels of IL-1β and IL-6 and increased plasma level of IL-10 in SHR rats (Figure 7).

### Effect of Exercise Training on RSNA

**Figure 8:**
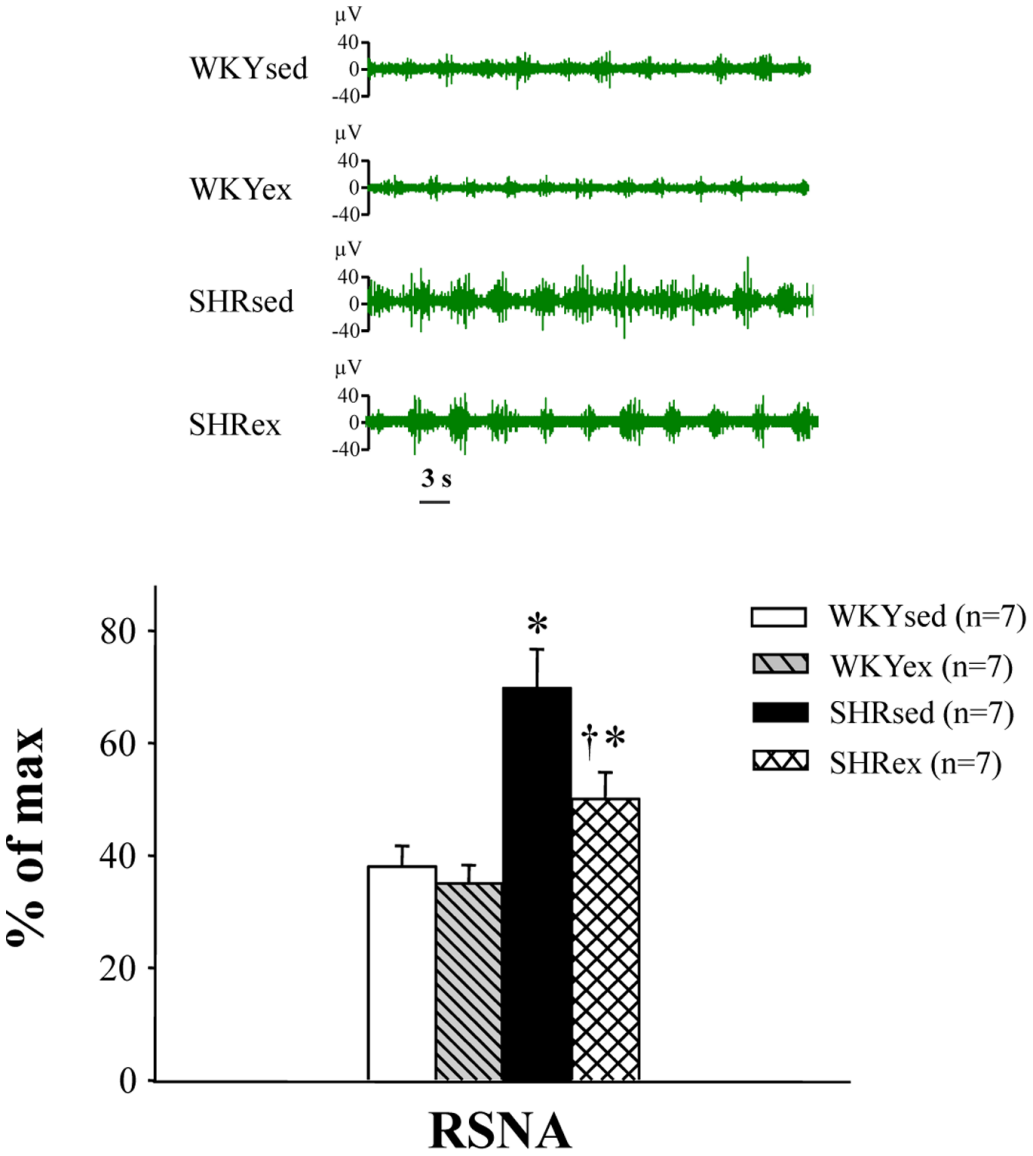
Effect of exercise training on renal sympathetic nerve activity (RSNA) in WKY and SHR rats. RSNA was increased in SHR rats compared with WKY rats. Exercise training decreased RSNA in SHR rats. *P<0.05 versus control (WKYsed or WKYex); † *P*<0.05 SHRex versus SHRsed.

RSNA was recorded 5 h after rats recovered from anesthesia. RSNA was increased in SHR rats compared with WKY rats. Exercise training decreased RSNA in SHR rats (Figure 8).

## Discussion

In this study, the role of exercise training on hypertension and cardiac hypertrophy was investigated. The novel findings of the present study are: (1) exercise training may attenuate hypertension-induced cardiac hypertrophy in rats by restoring the balances between the excitatory and inhibitory neurotransmitters and between pro- and anti-inflammatory cytokines in the PVN; (2) NF-κB p65 activity, oxidative stress and PICs in the PVN may be involved in the exercise-induced effects; (3) exercise training also has a beneficial effect by restoring the peripheral cytokine balance between pro- and anti-inflammatory cytokines in hypertension.

Elevated sympathetic outflow is commonly observed in hypertensive disorders and known to result in cardiac dysfunction and hypertrophy [2], [6]. Increasing evidence suggests changes of presympathetic neuronal activity in central nervous system might play a crucial role in blood pressure control, especially in the PVN [3], [16], [17]. The sympathetic outflow from the PVN depends on the balance of excitatory and inhibitory neurotransmitters in sympathetic neurons [18], [19]_._ Moreover, an imbalance among neurotransmitters caused by an overload of PICs in the PVN has been confirmed [16], [20]. Infusion of pro-inflammatory cytokine production inhibitor into the PVN causes the depression of the sympathetic activity along with the decreases in PVN levels of the excitatory neurotransmitters glutamate and NE as well as the increase in PVN level of inhibitory neurotransmitter GABA [21], [22]. In this study, we observed that SHR rats had exaggerated RSNA, higher PVN levels of glutamate, NE and PICs, and lower PVN level of GABA than WKY rats. These results indicate that the neurotransmitters and PICs in the PVN play important roles in sympathoexcitation and cardiac hypertrophy in hypertension.

Oxidative stress and the subsequent increase in the ROS production in the PVN have been proven to contribute to the progression of hypertension and cardiovascular disease [23]–[25]. In addition, a number of PICs have been identified to have the function of increasing ROS production [4], and the increased ROS in turn activates NF-κB and then results in the further increase in PICs production [26], [27]. Recent studies from our laboratory and others indicate inhibition of PICs production down-regulates NF-κB activity, gp91^phox^ expression and the free radical production in the PVN, and attenuates sympathoexcitation, suggesting the interaction among PICs, ROS, and NF-κB in heart failure [1], [28]_._ According to these findings, increased PICs in the PVN may be caused by oxidative stress and NF-κB activation.

Exercise training has been recommended as an important nonpharmacological treatment for hypertension [29]–[32]. In the present study, hypertension and cardiac hypertrophy as well as the sympathoexcitation in SHR rats were improved after 16 weeks of moderate ExT. These results were consistent with the findings from Agarwal and others that ExT exerts the anti-hypertensive effect [4], [33]–[35], but the detailed mechanisms of ExT on central nervous system have not been firmly established. Brain PICs and ROS were found to induce the imbalance between excitatory neurotransmitters glutamate and inhibitory neurotransmitters GABA in the PVN [18], [19], [28], [36]. The increased presynaptic glutamate release and over-expression of postsynaptic NMDA receptors have been confirmed to lead to the hyperactivity of the PVN neurons [37], [38]. On the contrary, the inhibitory neurotransmitter GABA in the PVN was obviously down-regulated in hypertensive rats. Chronic ExT not only attenuates PICs, alters the adrenergic and GABAergic system, and reduces oxidative stress, but also improves the anti-inflammatory mechanisms in the PVN and plasma. Therefore, these findings together with previous studies [4], [39]–[41] indicate that chronic exercise training attenuates hypertension and cardiac hypertrophy by restoring the balances between excitatory and inhibitory neurotransmitters and between pro- and anti-inflammatory cytokines in the PVN.

In summary, the results from this study indicates that: (1) hypertensive rats may have an imbalance between excitatory and inhibitory neurotransmitters within the PVN, and an imbalance between pro- and anti-inflammatory cytokines in the PVN, and accompanied by NF-κB p65 activation and oxidative stress in the PVN, and thereby may be responsible for sympathoexcitation, hypertensive response and cardiac hypertrophy; and (2) exercise training attenuates hypertension and cardiac hypertrophy by restoring the balance between the excitatory and inhibitory neurotransmitters and the balance between pro- and anti-inflammatory cytokines, and attenuating NF-κB p65 activity and oxidative stress in the PVN. Our findings provide further evidence and insight for the beneficial effect of exercise training on hypertension and cardiac hypertrophy.
